# Inter-individual Differences in Occipital Alpha Oscillations Correlate with White Matter Tissue Properties of the Optic Radiation

**DOI:** 10.1523/ENEURO.0224-19.2020

**Published:** 2020-04-07

**Authors:** Sorato Minami, Hiroki Oishi, Hiromasa Takemura, Kaoru Amano

**Affiliations:** 1 Center for Information and Neural Networks (CiNet), National Institute of Information and Communications Technology, and Osaka University, Suita 565-0871, Japan; 2 Graduate School of Frontier Biosciences, Osaka University, Suita 565-0871, Japan; 3 NTT Communication Science Laboratories, Atsugi 243-0198, Japan

**Keywords:** alpha oscillations, diffusion-weighted MRI, magnetoencephalography, neural oscillations, quantitative MRI, tissue property

## Abstract

Neural oscillations at ∼10 Hz, called alpha oscillations, are one of the most prominent components of neural oscillations in the human brain. In recent years, characteristics (power/frequency/phase) of occipital alpha oscillations have been correlated with various perceptual phenomena. However, the relationship between inter-individual differences in alpha oscillatory characteristics and the properties of the underlying brain structures, such as white matter pathways, is unclear. A possibility is that intrinsic occipital alpha oscillations are mediated by thalamocortical interaction; we hypothesized that the most promising candidate for characterizing the intrinsic alpha oscillation is optic radiation (OR), which is the geniculo-cortical pathway carrying signals between the lateral geniculate nucleus (LGN) and primary visual cortex (V1). We used resting-state magnetoencephalography (MEG) and diffusion-weighted/quantitative magnetic resonance imaging (MRI) (dMRI/qMRI) to correlate the frequency and power of occipital alpha oscillations with the tissue properties of the OR by focusing on the different characteristics across individuals. We found that the peak alpha frequency (PAF) negatively correlated with intracellular volume fraction (ICVF), reflecting diffusion properties in intracellular (axonal) space, whereas the peak alpha power was not correlated with any tissue properties measurements. No significant correlation was found between OR and beta frequency/amplitude or between other white matter tract connecting parietal and inferotemporal cortex and alpha frequency/amplitude. These results support the hypothesis that an interaction between thalamic nuclei and early visual areas is essential for the occipital alpha oscillatory rhythm.

## Significance Statement

Alpha oscillations, the most salient neural oscillations in the human brain, are known to be involved in various types of perception. The frequency of occipital alpha oscillations varies across participants, but the underlying structures regulating this variability remain unknown. We combined magnetoencephalography (MEG) measurements with diffusion-weighted MRI (dMRI) and quantitative MRI (qMRI) measurements and found that frequency properties of intrinsic occipital alpha oscillations correlated with a tissue property of the optic radiation (OR), a white matter tract connecting the lateral geniculate nucleus (LGN) and primary visual cortex (V1). This result supports the idea that thalamocortical interactions mediate the properties of intrinsic occipital alpha oscillations.

## Introduction

Alpha (8–13 Hz) oscillations in the human brain are closely related to several types of perceptual or cognitive functions. For example, the amplitude of intrinsic alpha oscillations is predictive of performance on a visual or memory task (Klimesch, 1999; Ergenoglu et al., 2004; Linkenkaer-Hansen et al., 2004; Thut et al., 2006; Palva and Palva, 2007; van Dijk et al., 2008; MacLean et al., 2012). In occipital alpha oscillations, MacLean et al. (2012) demonstrated a correlation between the inter-individual differences in resting-state occipital alpha power and attentional blink magnitude, which suggests that the amplitude of resting-state occipital alpha oscillations predicts attentional performance. Other more recent studies have linked the frequency of intrinsic occipital alpha oscillations to several types of perceptual phenomena such as the flickering wheel illusion (Sokoliuk and VanRullen, 2013), sound-induced double-flash illusion (Cecere et al., 2015), two-flash fusion (Samaha and Postle, 2015), and motion-induced spatial conflict (Minami and Amano, 2017). These studies imply that temporal resolution of visual processing depends in part on the rhythm of occipital alpha activity (Bonnefond et al., 2017). Thus, converging evidence suggests that occipital alpha oscillatory activity is a crucial substrate regulating the properties of human visual processing.

While the significance of occipital alpha oscillatory variations in visual processing has been established, how the neuroanatomical substrates underlying such variations correlate with them is not clear. Such variations can be related to individual differences in human brain structures, such as the white matter tracts supporting communication between distant brain areas (Catani and Thiebaut de Schotten, 2015). Since microstructural properties (e.g., myelination and axonal diameter) of white matter tracts significantly impact signal conductance, it has been hypothesized that white matter properties also relate to neural oscillatory activity (Fields, 2015). While a previous study reported a correlation between connectivity based on diffusion-weighted magnetic resonance imaging (dMRI) and alpha oscillations (Hindriks et al., 2015), it remains to be answered which white matter tract and what underlying microstructural properties may correlate with inter-individual variability in occipital alpha properties.

Other studies have investigated the source of occipital alpha oscillations electrophysiologically. One study (Bollimunta et al., 2011) reported that alpha current generators appeared to be in layer 4C of the primary visual cortex (V1), which receives inputs from the thalamic lateral geniculate nucleus (LGN), and in V1 layer 6, which projects back to the LGN. These findings suggest that the intrinsic occipital alpha oscillations are generated via interactions between LGN and V1. Using concurrent electroencephalography-functional MRI (EEG-fMRI) acquisition in humans, Liu et al. (2012) demonstrated that blood oxygenation level-dependent signals in visual thalamus (LGN and pulvinar) correlate with posterior alpha power, further supporting the importance of thalamo-cortical loop on a genesis of occipital alpha oscillation. Thus, we hypothesized that tissue properties of the optic radiation (OR), the main white matter tract that connects the LGN and V1, could underlie the inter-individual variability in occipital alpha oscillatory activity.

To test this hypothesis, we investigated whether the tissue properties of the OR are related to the frequency and/or power of the occipital alpha oscillations. We performed resting-state magnetoencephalography (MEG), dMRI, and quantitative MRI (qMRI) in healthy human participants. We quantified occipital alpha properties from the MEG data, and then identified the OR by analyzing dMRI data with constraints from prior anatomical knowledge (Sherbondy et al., 2008a). We evaluated the OR tissue properties using three metrics, namely the intracellular volume fraction (ICVF; Zhang et al., 2012) and orientation dispersion index (ODI; Zhang et al., 2012) estimated from dMRI and macromolecular tissue volume (MTV; Mezer et al., 2013) estimated from qMRI. We chose these metrics because previous studies have demonstrated their sensitivity for the different microstructural properties of white matter. ICVF and ODI are sensitive to different types of properties of axons such as volume and spatial configuration of axons, respectively (Mollink et al., 2017; Wang et al., 2019). In contrast, MTV is sensitive to lipid volume fractions in white matter, which correlate with myelin levels (Mezer et al., 2013; Duval et al., 2017). Inter-individual variability in these tissue properties of OR was compared with that in occipital alpha oscillations.

## Materials and Methods

### Participants

A total of 24 participants were recruited for the study (five females; 20–53 years, 25.7 ± 7.4 years, mean±SD). All participants had normal or corrected-to-normal vision and gave their written informed consent to participate. All experimental procedures were performed according to the Declaration of Helsinki and approved by the ethics committee of the National Institute of Information and Communications Technology (NICT). For any given participant, MEG and MRI scans were conducted on different days.

### Quantification and statistical analysis

#### MEG measurement and analyses

MEG data during the resting state were collected using a 360-channel whole head MEG system at Center for Information and Neural Networks (CiNet), NICT (NeuroMag 360, Elekta) comprising 204 planar gradiometers, 102 magnetometers, and 54 axial gradiometers. MEG signals were recorded at a sampling frequency of 1000 Hz. For the analysis, we used 204 planar gradiometers, which comprised two coils measuring spatial derivatives in orthogonal directions (*x* and *y*) of magnetic fields along the surface at 102 positions. Participants opened and closed their eyes for 30 s in response to a sound cue in a dark room. Each measurement (eyes-open or eyes-closed) was repeated six times for a total of 3 min for each resting condition.

Analyses of MEG data were performed using the FieldTrip toolbox (Oostenveld et al., 2011; http://www.fieldtriptoolbox.org/) running on MATLAB. We first removed artifacts originating from blinks or heartbeats by independent component analysis (James, 2002) and applied a 1- to 40-Hz bandpass filter. We then applied a fast Fourier transform to the data using 10-s time windows (10,000 time points) shifted by 1 s and averaged 126 spectra (21 spectra per each 30-s period). We then summed the power of the two gradient components at each of the 102 positions and selected five combined channels (pairs of planar gradiometers at five positions) with the largest alpha power for each participant. From these channels, we determined the peak alpha frequency (PAF) from the frequency showing the maximum power in the alpha band (defined as 8–13 Hz for this purpose).

As a control, we also estimated the peak beta frequency. Linear regression was applied to fit a linear model to the log-transformed spectrum in the beta range (13–30 Hz; Haegens et al., 2014); the fitted linear trend (the 1/f component) was subtracted from the spectrum because it obscures the smaller peaks in the beta range. Thereafter, we defined the peak beta frequency based on the frequency showing the maximum power in the beta band (13–30 Hz) for each participant using the subtracted spectrum.

Because the power of alpha/beta oscillations is highly susceptible to non-physiological factors such as the distance from the MEG helmet, we estimated the alpha/beta power in the source domain. For this purpose, we performed source localization using dynamic imaging of coherent sources (DICS; Gross et al., 2001). The DICS was applied at around the individual peak alpha/beta frequencies, and the neural activity index (NAI; Veen et al., 1997) was calculated. We averaged the NAI across the left/right superior, middle, and inferior occipital areas as an estimate of normalized occipital alpha/beta power in the resting-state. All regions of interest were anatomical defined based on the automated anatomical labeling (AAL) atlas (Tzourio-Mazoyer et al., 2002).

### Structural MRI data acquisition

#### Anatomical MRI data acquisition and tissue segmentation

T1-weighted magnetization prepared rapid gradient echo (MP-RAGE) images (1 mm isotropic; TR, 1900 ms; TE, 2.48 ms) were measured for all participants (*N *=* *24) using a 3T SIEMENS Prisma/Trio scanner at CiNet. An automated procedure in FreeSurfer software (https://surfer.nmr.mgh.harvard.edu/; Fischl, 2012) was used to determine the white/gray matter border that was used for subsequent dMRI analysis. The total acquisition time for the anatomical MRI data was ∼15 min for each participant.

#### dMRI data acquisition

dMRI data were measured from all participants (*N *=* *24) using a 3T SIEMENS Prisma scanner at CiNet with a 32-channel head coil. For data acquisition, dual-spin echoplanar imaging (EPI; TR, 3300 ms; TE, 66.4 ms; multiband factor, 3; partial Fourier, 5/8; voxel size, 2 × 2 × 2 mm^3^) was implemented in a multiband accelerated EPI pulse sequence provided by the Center for Magnetic Resonance Research, Department of Radiology, University of Minnesota (https://www.cmrr.umn.edu/multiband/; Setsompop et al., 2012).

Diffusion-weighted imaging with b = 300, 1000, and 2000 s/mm^2^ was conducted along 6, 30, and 64 isotropically distributed directions, respectively. Data were acquired with a pair of reversed-phase-encoding directions (A-P and P-A). In the dMRI session, eight non-diffusion-weighted (b = 0) images were acquired for each phase-encoding direction (A-P and P-A) to minimize EPI distortion. The total scan time for dMRI was ∼25 min for each participant.

#### qMRI data acquisition

qMRI data were measured from all participants (*N *=* *24) using a 3T SIEMENS Trio scanner at CiNet with a 32-channel head coil. Parameters for qMRI were as described in a previous publication (Mezer et al., 2013). Four fast low-angle shot (FLASH) images were measured with flip angles of 4°, 10°, 20°, and 30° (TR, 12 ms; TE, 2.41 ms) and a scan resolution of 1 mm isotropic. Five additional spin echo inversion recovery (SEIR) scans were also measured with an EPI readout (TR, 3 s; TE, 49 ms; 2× acceleration) to remove field inhomogeneities. The inversion times were 50, 200, 400, 1200, and 2400 ms. In-plane resolution and slice thickness of the additional scan were 2 × 2 mm^2^ and 4 mm, respectively. The total scan time of qMRI was ∼35 min for each participant.

### dMRI data analysis

#### Preprocessing

dMRI images were corrected for susceptibility-induced distortions using FSL TOPUP tools (Andersson et al., 2003). Eddy current distortions and participant motion in the dMRI images were corrected using FSL EDDY tools (Andersson and Sotiropoulos, 2016). Finally, dMRI images were aligned to the T1-weighted MP-RAGE images using mrDiffusion tools implemented in Vistasoft (https://github.com/vistalab/vistasoft).

#### Estimation of ICVF and ODI

After preprocessing, a neurite orientation dispersion and density imaging (NODDI) model was fitted to dMRI data using the NODDI MATLAB toolbox (http://mig.cs.ucl.ac.uk/index.php?n=Tutorial.NODDImatlab) to obtain ICVF and ODI maps in individual participants (Zhang et al., 2012). ICVF is a marker of neuronal density, with a high value indicating densely packed fibers, while ODI quantifies the coherence of fiber orientations, with a low value indicating aligned fibers.

#### Tractography on the OR

The OR was identified using a probabilistic tractography method, ConTrack (Sherbondy et al., 2008b), which is known to have sufficient sensitivity to identify the OR from dMRI data (Sherbondy et al., 2008a). First, the approximate location of the LGN was estimated by manual inspection of the T1-weighted image and deterministic tractography from the optic chiasm (Ogawa et al., 2014). Then, an 8-mm radius sphere that covered the LGN endpoints of streamlines from the optic chiasm was placed. Second, the location of V1 was identified using a probabilistic atlas of retinotopic visual areas (Wang et al., 2015). Using ConTrack, we generated 100,000 candidate streamlines connecting LGN and V1 (angle threshold, 90°; step size, 1 mm). Tracking was restricted using the white matter mask generated by tissue segmentation. Streamlines passing through ventricles were also rejected. Finally, the top 30,000 streamlines with the highest scores in ConTrack (Sherbondy et al., 2008b) were selected from candidate streamlines for subsequent analyses. Further details on the methods to identify the OR using ConTrack are described elsewhere (Ogawa et al., 2014; Takemura et al., 2017, 2019; Oishi et al., 2018).

#### Tractography on the posterior arcuate fasciculus (pArc)

As an anatomical control, we also analyzed the pArc (Catani et al., 2005; Weiner et al., 2017). Since previous studies showed that it is essential to use a tractography algorithm with better sensitivity for resolving crossing fibers for pArc (Weiner et al., 2017), we used a multi-shell, multi-tissue constrained spherical deconvolution (CSD; *L_max_* = 8; Jeurissen et al., 2014) to estimate fiber orientation distribution in each voxel with MRtrix3 (http://www.mrtrix.org/; Tournier et al., 2012). We then used CSD-based probabilistic tractography implemented in MRtrix3 to generate 2 million streamlines for each dMRI dataset (step size = 1 mm; maximum angle between successive steps = 45°; minimum length = 4 mm; maximum length = 250 mm; Fiber orientation distribution amplitude stopping criterion = 0.05). The seed voxels for tracking were randomly chosen from the entire white matter mask. Finally, the pArc was identified from whole-brain streamlines using automated pipelines implemented as a part of the AFQ toolbox (https://github.com/yeatmanlab/AFQ/tree/master/vof; Yeatman et al., 2014; Weiner et al., 2017).

#### Across-session averaging and outlier exclusion

Each streamline of the identified white matter tracts (OR and pArc) was merged from two dMRI sessions with reversed-phase-encoding directions. Outlier streamlines were excluded based on criteria used in previous studies (Takemura et al., 2017; Oishi et al., 2018) for subsequent evaluation of tissue properties.

#### Estimation of tract length

Furthermore, we estimated the tract length of the OR in each participant by calculating the mean length of the streamlines belonging to the OR. The tract lengths in the left and right OR were averaged.

### qMRI data analysis

Using the mrQ software package (https://github.com/mezera/mrQ) in MATLAB, both the FLASH and SEIR scans were processed to produce the MTV maps (Mezer et al., 2013). MTV quantifies the tissue volume density by estimating a quantitative proton-density map from the qMRI dataset after correcting for RF coil bias by the mrQ analysis pipeline using SEIR-EPI scans (Barral et al., 2010; Mezer et al., 2013). Because the CSF voxels are entirely filled with water, we assumed that these voxels had a full water volume fraction (WVF). We then calculated the WVF ratio in cortical gray or white matter voxels compared with CSF. MTV was defined as: MTV = 1 − WVF. It was used to quantify the non-proton macromolecule volume fraction in each voxel. Finally, the MTV maps were aligned to T1-weighted MP-RAGE images to register them with the dMRI data. The full analysis pipeline can be found in previous publications (Mezer et al., 2013, 2016). Because a large fraction of macromolecules in white matter is myelin, previous works have suggested that MTV is a relatively sensitive metric for myelin levels (Duval et al., 2017; Berman et al., 2018). Therefore, we tested MTV as a metric providing a different type of microstructural information compared with ICVF and ODI, which are considered to reflect the volume or spatial configuration of axons.

As a supplemental analysis, we also estimated g-ratio that is defined as the ratio between the inner and outer diameters of the myelin sheath and can be estimated from dMRI and qMRI data (Stikov et al., 2015; Ellerbrock and Mohammadi, 2018; Berman et al., 2019). We calculated the putative g-ratio in each voxel by combining MTV and NODDI measurements following a formula proposed by a previous study (Berman et al., 2019).

### Evaluating the tissue property of white matter tracts

Tissue measurements (ICVF, ODI, and MTV) of each visual white matter tract were evaluated using the methods used in previous studies (Yeatman et al., 2012; Duan et al., 2015). Briefly, each streamline was resampled to 100 equidistant nodes. ICVF, ODI, and MTV were calculated at each node of each streamline. The property at each node was then summarized by taking a weighted average of the tissue measurements (ICVF, ODI, and MTV) on individual streamlines within that node. The weight of each streamline was assigned based on the Mahalanobis distance from the tract core. The first and last ten nodes close to the gray-white matter interface, where the tract is likely to be heavily intersected by the superficial U-fiber system, were excluded. We then averaged 80 values along different nodes in the OR for each tissue measurement to obtain a single-number summary of participant-specific ICVF, ODI, and MTV. Finally, these tissue measurements were averaged across hemispheres for each tract. Since resting-state occipital alpha oscillations are not restricted to a specific hemisphere, it is reasonable to compare it with white matter tract properties averaged across hemispheres.

#### Statistical comparisons

We calculated Pearson correlation coefficients between the frequency/amplitude (NAI) of alpha oscillations in the resting condition and the MRI-based tissue properties of the white matter tracts for each participant (uncorrected *p*-values are described in the text). We applied a Bonferroni correction to the six comparisons between two MEG measures (PAF/NAI) and three tissue properties (ICVF/ODI/MTV) by adjusting the threshold for statistical significance to *alpha* = 0.0083.

We also conducted a default Bayesian hypothesis test for the presence of correlations using the BayesMed package in R (Wetzels and Wagenmakers, 2012). Bayes factor values (*BF*_10_) were calculated to assess the relative plausibility of the observed data under the two competing hypotheses (Jeffreys, 1961; Wetzels and Wagenmakers, 2012; *BF*_10_ between 3 and 10 indicates substantial evidence for the alternative hypothesis; *BF*_10_ between 1 and 3 provides anecdotal evidence for the alternative hypothesis; *BF*_10_ between 0.33 and 1 indicates anecdotal evidence for the null hypothesis; and *BF*_10_ between 0.1 and 0.33, substantial evidence for the null hypothesis).

To assess the specificity of the correlation between alpha oscillations and OR, we calculated the correlation for another frequency band and for another white matter tract and tested the difference between the two dependent correlations with one variable in common (Lee and Preacher, 2013).

## Results

### Relationship between alpha oscillations and the OR

**Figure 1.**
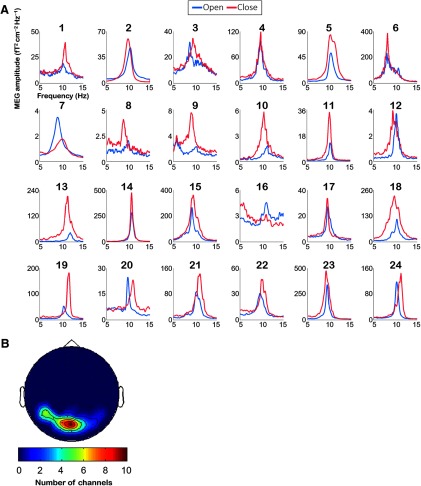
Power spectra of MEG data for all participants and distributions map of selected channels averaged across participants. ***A***, The power spectra of the eyes-open and eyes-closed resting conditions for all participants. Ten-second moving time windows (10,000 time points) were used for the first Fourier transform (FFT) analysis. ***B***, The channels used for the FFT analysis. Five combined channels (10 planar gradiometers) showing the largest alpha power were selected for each participant and were then pooled across participants. Distribution maps indicate the total selected number of combined channels at each location. Selected channels cover the parieto-occipital area.

Figure 1 shows the MEG spectra of all participants in the eyes-open and eyes-closed resting conditions measured from 10 planar gradiometers showing the maximum amplitude. Averaged PAF values in the eyes-open and eyes-closed resting conditions were ∼10 Hz (eyes-open: 10.2 ± 0.2 Hz; eyes-closed: 10.1 ± 0.2 Hz, mean ± SE). No statistically significant difference was observed between PAF values in the eyes-open and eyes-closed conditions (*t*_(23)_ = 0.99, *p *=* *0.33, *BF*_10_ = 0.33, two-tailed paired *t* test). In addition, NAI values in the eyes-open condition, calculated by performing source localization, were not significantly different from those in the eyes-closed condition (eyes-open: 6.03 ± 0.65; eyes-closed: 6.87 ± 0.72, mean ± SE *t*_(23)_ = 0.87, *p *=* *0.39, *BF*_10_ = 0.39, two-tailed paired *t* test).

**Figure 2.**
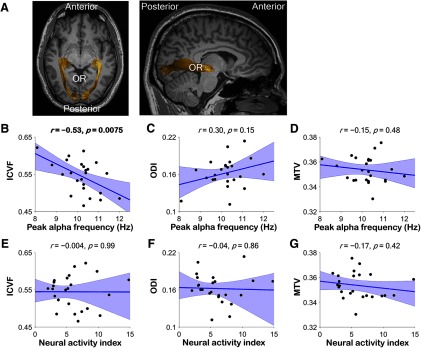
Relationship between the alpha frequency/amplitude and tissue properties of the OR. ***A***, The OR identified by tractography in a representative participant (left, axial view; right, sagittal view). ***B–D***, Correlation between the peak alpha frequency (PAF) in the eyes-open resting condition and the ICVF (***B***), ODI (***C***), or MTV (***D***) of OR for all participants (*N *=* *24). The negative correlation between the PAF and the ICVF was significant. ***E–G***, Correlation between the neural activity index (NAI) at the PAF in the eyes-open resting condition and the ICVF (***E***), ODI (***F***), or MTV (***G***) of OR. The NAI and the ICVF/ODI/MTV were not significantly correlated.

Figure 2*A* shows the trajectory of the OR identified by probabilistic tractography on the dMRI dataset. We compared PAF (*N *=* *24; Fig. 2*B–D*) and NAI (*N *=* *24; Fig. 2*E–G*) in the eyes-open resting condition with the ICVF, ODI, and MTV in the OR. We found a significant negative correlation between the PAF and ICVF (*r* = −0.53, *p *=* *0.0075; Fig. 2*B*) but not between the PAF and ODI/MTV [ODI, *r *=* *0.30, *p *=* *0.15 (Fig. 2*C*); MTV, *r* = −0.15, *p *=* *0.48 (Fig. 2*D*)] in the OR. The Bayes factor values supported the results of this conventional statistical analysis (*BF*_10_ = 4.63, 0.43, and 0.20 for ICVF, ODI, and MTV, respectively). There was no significant correlation between NAI and ICVF/ODI/MTV in the OR [ICVF, *r* = −0.004, *p *=* *0.99, *BF*_10_ = 0.16 (Fig. 2*E*); ODI, *r* = −0.04, *p *=* *0.86, *BF*_10_ = 0.16 (Fig. 2*F*); MTV, *r* = −0.17, *p *=* *0.42, *BF*_10_ = 0.22 (Fig. 2*G*)].

Similar to the findings from the eyes-open condition, the ICVF in the OR was negatively correlated with PAF in the eyes-closed resting condition (*r* = −0.42, *p *=* *0.028, *BF*_10_ = 1.66), but this effect was not statistically significant after Bonferroni correction. The correlation between the PAF and ODI/MTV in the OR was not significant (ODI, *r* = −0.07, *p *=* *0.75, *BF*_10_ = 0.17; MTV, *r *=* *0.12, *p *=* *0.57, *BF*_10_ = 0.18), and there was no significant correlation between the NAI and ICVF/ODI/MTV in the OR (ICVF, *r* = −0.13, *p *=* *0.55, *BF*_10_ = 0.19; ODI, *r* = −0.18, *p *=* *0.41, *BF*_10_ = 0.22; MTV, *r* = −0.37, *p *=* *0.078, *BF*_10_ = 0.73).

To investigate whether the relationship between PAF in the eyes-open resting condition and ICVF holds even within individuals, we compared the PAF at the hemisphere with smaller ICVF with that at the hemisphere with larger ICVF. However, no significant difference in PAF was observed (*t*_(23)_ = 0.90, *p *=* *0.37, *BF*_10_ = 0.40, two-tailed paired *t* test). This is most likely because PAF highly correlated between the hemispheres (*r *=* *0.99, *p *<* *0.0001, *BF*_10_ = 3e^15^).

### Relationship between beta oscillations and OR

**Figure 3.**
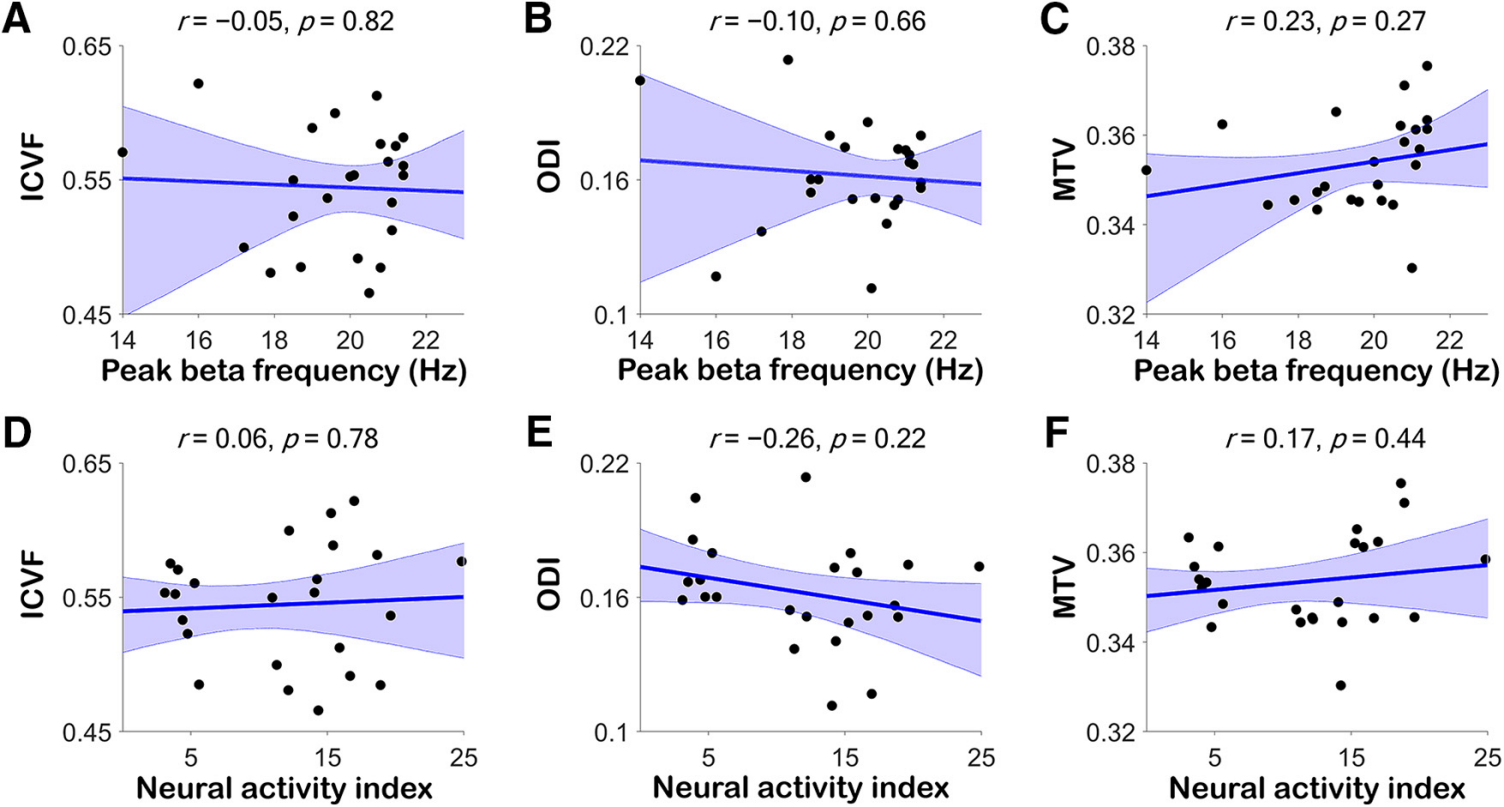
Relationship between the beta frequency/amplitude and tissue properties of the OR. ***A–C***, Correlation between the peak beta frequency in the eyes-open resting condition and the ICVF (***A***), ODI (***B***), or MTV (***C***) of the OR for all participants (*N *=* *24). ***D–F***, Correlation between the NAI at the peak beta frequency in the eyes-open resting condition and the ICVF (***D***), ODI (***E***), or MTV (***F***) of OR. None of the correlations were significant.

To test whether the correlation between the characteristics of neural oscillations and tissue properties in the OR is specific to the alpha band, we performed a control analysis to compare the peak frequency/NAI of beta oscillations and OR properties (in the eyes-open resting condition). Consequently, neither the peak frequency [ICVF, *r* = −0.05, *p *=* *0.82, *BF*_10_ = 0.16 (Fig. 3*A*); ODI, *r* = −0.10, *p *=* *0.66, *BF*_10_ = 0.17 (Fig. 3*B*); MTV, *r *=* *0.23, *p *=* *0.27, *BF*_10_ = 0.29 (Fig. 3*C*)] nor NAI [ICVF, *r *=* *0.06, *p *=* *0.78, *BF*_10_ = 0.16 (Fig. 3*D*); ODI, *r* = −0.26, *p *=* *0.22, *BF*_10_ = 0.33 (Fig. 3*E*); MTV, *r *=* *0.17, *p *=* *0.44, *BF*_10_ = 0.21 (Fig. 3*F*)] was correlated with OR properties.

### Relationship between alpha oscillations and pArc

**Figure 4.**
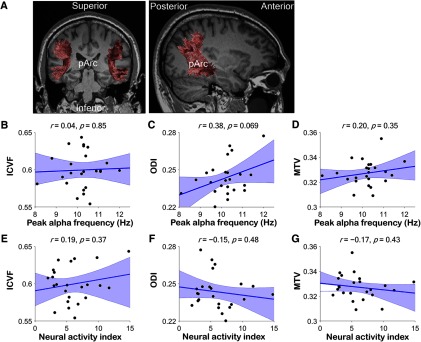
Relationship between the peak alpha frequency/amplitude and tissue properties of the pArc. ***A***, pArc identified using tractography in a representative participant (left, coronal view; right, sagittal view). ***B–D***, Correlation between PAF in the eyes-open resting condition and the ICVF (***B***), ODI (***C***), or MTV (***D***) of pArc for all participants (*N *=* *24). ***E–G***, Correlation between the NAI at the PAF in the eyes-open resting condition and the ICVF (***E***), ODI (***F***), or MTV (***G***) of pArc. None of the correlations were significant.

As an anatomical control, we assessed correlations between alpha oscillatory characteristics and ICVF/ODI/MTV of the pArc (Fig. 4*A*) in the eyes-open resting condition. pArc tissue properties were not significantly correlated with PAF [ICVF, *r *=* *0.04, *p *=* *0.85, *BF*_10_ = 0.16 (Fig. 4*B*); ODI, *r *=* *0.38, *p *=* *0.069, *BF*_10_ = 0.81 (Fig. 4*C*); MTV, *r *=* *0.20, *p *=* *0.35, *BF*_10_ = 0.24 (Fig. 4*D*)] or NAI [ICVF, *r *=* *0.19, *p *=* *0.37, *BF*_10_ = 0.23 (Fig. 4*E*); ODI, *r* = −0.15, *p *=* *0.48, *BF*_10_ = 0.20 (Fig. 4*F*); MTV, *r* = −0.17, *p *=* *0.43, *BF*_10_ = 0.21 (Fig. 4*G*)].

### Specificity of relationship between PAF and ICVF of OR

To support the specificity of the correlation between the PAF and ICVF of OR, we tested whether this correlation is significantly different from the correlation for a different frequency band (beta) and from the correlation for a different white matter tract (pArc). The correlation between PAF in the eyes-open resting condition and ICVF of OR was significantly stronger than the correlation between the peak beta frequency and ICVF of OR (*z* = −2.30, *p *=* *0.021, two-tailed test) and also from the correlation between PAF and ICVF of pArc (*z* = −2.73, *p *=* *0.006, two-tailed test). These results suggest that the correlation between neural oscillations and ICVF is specific to the relationship between alpha oscillations and OR.

### Relationship between PAF and other tissue properties of OR

As a supplementary analysis, we tested whether the significant correlation between the PAF and ICVF in the OR (Fig. 2*B*) is explained by other metrics of structural properties such as MRI-based estimation of g-ratio (Berman et al., 2019) and tract length. We assessed the correlations between PAF and these variables of the OR in the eyes-open resting condition. As a result, g-ratio and length did not significantly correlate with PAF (g-ratio, *r *=* *0.13, *p *=* *0.55, *BF*_10_ = 0.18; length, *r* = −0.24, *p *=* *0.26, *BF*_10_ = 0.20), suggesting that the properties of occipital alpha oscillations might not be related with these metrics.

## Discussion

Inter-individual differences in occipital alpha oscillations are reflected in several aspects of perception and behavior (Klimesch, 1999; MacLean et al., 2012; Sokoliuk and VanRullen, 2013; Cecere et al., 2015; Samaha and Postle, 2015; Minami and Amano, 2017). In the current study, we found that the inter-individual differences in PAF were significantly correlated with the ICVF of the OR. We also found this correlation to be absent in the control conditions. Beta-band frequencies were not significantly correlated by any tissue properties in the OR. Tissue properties of the pArc, an association fiber tract located posteriorly to the lateral sulcus and connecting the parietal and inferotemporal cortices (Catani et al., 2005; Martino and García-Porrero, 2013; Weiner et al., 2017; Panesar et al., 2019), were not correlated with any alpha band oscillatory characteristics. Importantly, correlation between PAF and the ICVF of the OR was significantly stronger than that in these control analyses for beta oscillations and pArc, confirming the specificity of the correlation.

We employed probabilistic tractography to identify the trajectory of the OR (Fig. 2*A*; see also Materials and Methods); we used dMRI- and qMRI-based microstructural measurements to study the OR tract’s properties. We chose to analyze ICVF and ODI, which are indexes of intracellular (axonal) volume of white matter and the degree of spread of fiber trajectories, respectively (Zhang et al., 2012). We further chose to analyze MTV, which quantifies the non-proton (non-water) neural tissue density (Mezer et al., 2013). These three metrics have been evaluated by a number of investigators and data describing their relationships with underlying microstructural properties and their reliability as analytical measures have been published previously (Zhang et al., 2012; Mezer et al., 2013; Duval et al., 2017; Mollink et al., 2017; Berman et al., 2018; Fukutomi et al., 2018; McCunn et al., 2019; Wang et al., 2019). Thus, we used these three metrics, which are sensitive to different properties of white matter tissue that are independent of each other, to investigate what structural factors are associated with alpha oscillation. We confirmed that there were no significant correlations among ICVF, ODI, and MTV in OR (ICVF and MTV, *r *=* *0.32, *p *=* *0.12, *BF*_10_ = 0.52; ODI and MTV, *r* = −0.05, *p *=* *0.81, *BF*_10_ = 0.16; ICVF and ODI, *r* = −0.09, *p *=* *0.66, *BF*_10_ = 0.17), supporting independence among these three metrics.

In the present study, the PAF negatively correlated with ICVF of the OR. ICVF is an index to quantify the diffusion signal in intracellular space and to estimate volume of axonal space in white matter (Zhang et al., 2012). A previous study validated the use of ICVF by showing that ICVF is correlated with histologic measurements of neurite density (Wang et al., 2019). In the present study, we found no significant correlation between PAF and MTV of OR, which is sensitive to lipid volume fractions and may reflect myelin levels in the white matter (Mezer et al., 2013; Berman et al., 2018; Duval et al., 2017). Therefore, our results suggest that the volume of axons, rather than myelin levels, might be associated with inter-individual differences in occipital PAF. However, we emphasize that the microstructural interpretation of NODDI parameters (ICVF/ODI) is still actively debated among investigators; indeed, it is not yet clear how much the histologic validation of MRI metric by a single study can be generalized to measurements in other areas or datasets collected using different resolutions or acquisition parameters (Harkins et al., 2016; Jelescu et al., 2016; Schilling et al., 2018; Wang et al., 2019). Further understanding of the correlation described in this study requires future investigations that clarify the relationship between MRI-based measurements and microstructure of the OR.

Although we found a correlation between inter-individual differences in alpha oscillations and the OR, the causal relationship between them remains to be explored. Recent studies have demonstrated that white matter plasticity is influenced by properties of signal conduction along axons or learning (Fields, 2015; Wake et al., 2015; Sampaio-Baptista and Johansen-Berg, 2017). One might hypothesize that conductance of oscillatory neuronal activities, such as alpha oscillations, may affect such plasticity. Conversely, a recent review on neural network models (Pajevic et al., 2014) proposed that the variations in conduction velocity and conduction delay relating to the white matter microstructure significantly change the interaction between two coupled oscillators, leading to profound effects on the oscillation amplitude and frequency. Thus, we speculate that occipital alpha oscillations and OR structural properties exert influences on each other. In other words, information transmission between the LGN and V1 via alpha oscillations may change the microstructural properties of the OR, whereas the microstructural properties of the OR may also modulate the alpha oscillations.

A previous study by Bollimunta et al. (2011) indicated that intrinsic alpha activities are generated in layers 4C and 6 of V1, which are interconnected with the LGN. This would suggest that bidirectional thalamocortical neurotransmission shapes the activity of the alpha band neural oscillators. In addition, simultaneous local field potential measurements in the LGN and V1 and directed connectivity analysis suggested that feedback processing from V1 to LGN is mediated by the alpha oscillations (Bastos et al., 2014). These physiological findings relating alpha band activity to the LGN-V1 loop are consistent with ours. However, we cannot rule out the involvement of other corticothalamic loops in alpha oscillations because MRI resolution might be insufficient to distinguish LGN-V1 fibers from the fibers between other nuclei (e.g., the pulvinar; Bridge et al., 2016) and V1 if they overlapped within the same voxels. In fact, a simultaneous EEG-fMRI study demonstrated that blood oxygenation level-dependent signals in LGN and pulvinar correlate with posterior alpha power, suggesting a possibility that alpha oscillation is generated by multiple thalamo-cortical loops including those in pulvinar (Liu et al., 2012).

Some previous studies have examined the relationship between properties of occipital alpha oscillation and measurements of white matter (Hindriks et al., 2015; Renauld et al., 2016). Hindriks et al. (2015) performed tractography on dMRI data and counted the number of streamlines connecting the areas defined by the AAL atlas (Tzourio-Mazoyer et al., 2002). They reported that the amplitude of the occipital alpha oscillations correlated with the streamline counts on the fiber pathways between the V1 and other visual areas. Unlike the study herein, they did not focus on a specific white matter tract. That may be one of the reasons why they found a correlation between the alpha power and white matter properties while we did not. The previous study also focused only on the alpha power, whereas the current study included alpha frequency, which is also crucial for characterizing visual processing (Sokoliuk and VanRullen, 2013; Cecere et al., 2015; Samaha and Postle, 2015; Minami and Amano, 2017). Renauld et al. (2016) did not find significant correlation between OR tissue properties and PAF, which were found in the present study. They used fractional anisotropy and apparent fiber density, which are the metrics on tissue properties that were different from those used in our study. They estimated these metrics from single-shell dMRI data (b = 1000 s/mm^2^, 64 directions) acquired using a 1.5-T MRI. In contrast, we performed analysis on NODDI metrics (ICVF) based on multi-shell (b = 300, 1000, and 2000 s/mm^2^) dMRI data with larger number of directions (100 directions in total) acquired by 3-T MRI with a strong gradient (80 mT/m). We speculate that different conclusions arise at least partly because of the higher sensitivity and specificity of our measurements, derived from differences in dMRI signal modeling, acquisition scheme, and hardware.

In summary, we found that the frequency of the intrinsic occipital alpha oscillations was negatively correlated with the microstructural property (ICVF) of the OR, suggesting the OR may serve as an essential anatomical substrate of intrinsic alpha oscillations. Currently, there is no clear interpretation to explain the underlying mechanism for these correlations. Future studies will be required to clarify the relationships between ICVF and the microstructure of white matter tracts.

## Synthesis

Reviewing Editor: Bradley Postle, University of Wisconsin Decisions are customarily a result of the Reviewing Editor and the peer reviewers coming together and discussing their recommendations until a consensus is reached. When revisions are invited, a fact-based synthesis statement explaining their decision and outlining what is needed to prepare a revision will be listed below. The following reviewer(s) agreed to reveal their identity: Tom Marshall, Aviv Mezer.

R1

The authors have provided clear and thorough revisions that have substantially improved the quality of the manuscript.

I have only one further minor comment and do not need to see the manuscript again. The authors now provide Bayes factor estimations for all statistical tests, but missed them out for the t-tests comparing eyes closed and eyes open alpha (lines 261-263). I suspect this is an oversight but would appreciate the authors reporting BF here too for consistency.

R2

To claim for specificity, the authors report non-significant effects in both control conditions. While this reasoning is prevalent in the neuroimaging studies, it is flawed and does not support claims for specificity. This is explained in detail in the paper “Erroneous analyses of interactions in neuroscience: a problem of significance” (Nieuwenhuis et al., Nature Neuroscience, 2011). Instead of reporting the different in p-values (one is significant, and the other is not), the underlying effects themselves must be compared. This can be done in various ways, such as an ANOVA analysis.

Methods:

In the Methods, the authors write that the peak frequency in the beta band was searched in the range 15-25 Hz (and not 13-30 Hz, as they defined the beta frequency). In contrast, in Figure 2, it seems that the 14 Hz frequency was also included. Please correct the text to the actual range that was used in the analysis.

The control analysis:

To support the specificity of their claims, the authors conducted two additional analyses - one for a different frequency band (beta) and one for a different anatomical structure (the pArc). This is indeed an important analysis that strengthens the manuscript.

Nevertheless, the statistical analysis of the results requires improvement. To claim for specificity, the authors report non-significant effects in both control conditions. While this reasoning is prevalent in the neuroimaging studies, it is flawed and does not support claims for specificity. This is explained in detail in the paper “Erroneous analyses of interactions in neuroscience: a problem of significance” (Nieuwenhuis et al., Nature Neuroscience, 2011). Instead of reporting the different in p-values (one is significant, and the other is not), the underlying effects themselves must be compared. This can be done in various ways, such as an ANOVA analysis.

Also, the reader can use a short explanation of why the beta band is a control.

Suggestions:

To study the inter-subject variability in properties of alpha oscillations with relation to dMRI and qMRI measurements, the authors averaged the measurements across hemispheres. Since there is literature on laterality effects in the posterior alpha oscillations, the authors could consider performing an intra-subject analysis, to see if the results found at the group level also hold within individuals.

Since they discuss conduction, and they have MTV and NODDI, they could potentially try using g-ratio for the prediction. More importantly, they should try and test the contribution of the OR length.

Additional relevant references:

Please also refer to the paper “Semi-Automatic Segmentation of Optic Radiations and LGN, and Their Relationship to EEG Alpha Waves” (Renauld et al., 2016), in which the authors compared diffusivity measures in the OR to alpha power and peak alpha frequency, as measured with EEG,

https://www.ncbi.nlm.nih.gov/pmc/articles/PMC3472152/

You may refer to “Finding Thalamic BOLD Correlates to Posterior Alpha EEG” (Liu et al., 2012), in which the authors find that the visual thalamus, and the pulvinar, in particular, is involved in the generation of posterior alpha oscillations.

Minor:

In the Abstract: help mediate -> mediate

In the caption of figure 1: Distributions maps -> Distributions map.

In the Discussion: In the current study, the PAF was correlated (albeit negatively) -> In the current study, the PAF was (negatively) correlated

In the Discussion:

which implies that feedback processing from V1 to LGN is performed in the alpha band. ->

which implies that feedback processing from V1 to LGN is mediated by the alpha band.

## References

[B1] Andersson JL, Skare S, Ashburner J (2003) How to correct susceptibility distortions in spin-echo echo-planar images: application to diffusion tensor imaging. Neuroimage 20:870–888. 10.1016/S1053-8119(03)00336-7 14568458

[B2] Andersson JLR, Sotiropoulos SN (2016) An integrated approach to correction for off-resonance effects and subject movement in diffusion MR imaging. Neuroimage 125:1063–1078. 10.1016/j.neuroimage.2015.10.019 26481672 PMC4692656

[B3] Barral JK, Gudmundson E, Stikov N, Etezadi-Amoli M, Stoica P, Nishimura DG (2010) A robust methodology for in vivo T1 mapping. Magn Reson Med 64:1057–1067. 10.1002/mrm.22497 20564597 PMC2962940

[B4] Bastos AM, Briggs F, Alitto HJ, Mangun GR, Usrey WM (2014) Simultaneous recordings from the primary visual cortex and lateral geniculate nucleus reveal rhythmic interactions and a cortical source for gamma-band oscillations. J Neurosci 34:7639–7644. 10.1523/JNEUROSCI.4216-13.2014 24872567 PMC4035524

[B5] Berman S, West KL, Does MD, Yeatman JD, Mezer AA (2018) Evaluating g-ratio weighted changes in the corpus callosum as a function of age and sex. Neuroimage 182:304–313. 10.1016/j.neuroimage.2017.06.076 28673882 PMC5748016

[B6] Berman S, Filo S, Mezer AA (2019) Modeling conduction delays in the corpus callosum using MRI-measured g-ratio. Neuroimage 195:128–139. 10.1016/j.neuroimage.2019.03.025 30910729

[B7] Bollimunta A, Mo J, Schroeder CE, Ding M (2011) Neuronal mechanisms and attentional modulation of corticothalamic α oscillations. J Neurosci 31:4935–4943. 10.1523/JNEUROSCI.5580-10.2011 21451032 PMC3505610

[B8] Bonnefond M, Kastner S, Jensen O (2017) Communication between brain areas based on nested oscillations. eNeuro 4:ENEURO.0153-16.2017. 10.1523/ENEURO.0153-16.2017 PMC5367085 28374013

[B9] Bridge H, Leopold DA, Bourne JA (2016) Adaptive pulvinar circuitry supports visual cognition. Trends Cogn Sci 20:146–157. 10.1016/j.tics.2015.10.003 26553222 PMC4724498

[B10] Catani M, Jones DK, Ffytche DH (2005) Perisylvian language networks of the human brain. Ann Neurol 57:8–16. 10.1002/ana.20319 15597383

[B11] Catani M, Thiebaut de Schotten M (2015) Atlas of human brain connections. New York: Oxford University Press.

[B12] Cecere R, Rees G, Romei V (2015) Individual differences in alpha frequency drive crossmodal illusory perception. Curr Biol 25:231–235. 10.1016/j.cub.2014.11.034 25544613 PMC4300399

[B13] Duan Y, Norcia AM, Yeatman JD, Mezer A (2015) The structural properties of major white matter tracts in strabismic amblyopia. Invest Ophthalmol Vis Sci 56:5152–5160. 10.1167/iovs.15-17097 26241402 PMC4525637

[B14] Duval T, Le Vy S, Stikov N, Campbell J, Mezer A, Witzel T, Keil B, Smith V, Wald LL, Klawiter E, Cohen-Adad J (2017) g-Ratio weighted imaging of the human spinal cord in vivo. Neuroimage 145:11–23. 10.1016/j.neuroimage.2016.09.018 27664830 PMC5179300

[B15] Ellerbrock I, Mohammadi S (2018) Four in vivo g‐ratio‐weighted imaging methods: comparability and repeatability at the group level. Hum Brain Mapp 39:24–41. 10.1002/hbm.23858 29091341 PMC6866374

[B16] Ergenoglu T, Demiralp T, Bayraktaroglu Z, Ergen M, Beydagi H, Uresin Y (2004) Alpha rhythm of the EEG modulates visual detection performance in humans. Brain Res Cogn Brain Res 20:376–383. 10.1016/j.cogbrainres.2004.03.009 15268915

[B17] Fields RD (2015) A new mechanism of nervous system plasticity: activity-dependent myelination. Nat Rev Neurosci 16:756–767. 10.1038/nrn4023 26585800 PMC6310485

[B18] Fischl B (2012) FreeSurfer. Neuroimage 62:774–781. 10.1016/j.neuroimage.2012.01.021 22248573 PMC3685476

[B19] Fukutomi H, Glasser MF, Zhang H, Autio JA, Coalson TS, Okada T, Togashi K, Van Essen DC, Hayashi T (2018) Neurite imaging reveals microstructural variations in human cerebral cortical gray matter. Neuroimage 182:488–499. 10.1016/j.neuroimage.2018.02.017 29448073 PMC6326835

[B20] Gross J, Kujala J, Hamalainen M, Timmermann L, Schnitzler A, Salmelin R (2001) Dynamic imaging of coherent sources: studying neural interactions in the human brain. Proc Natl Acad Sci USA 98:694–699. 10.1073/pnas.98.2.694 11209067 PMC14650

[B21] Haegens S, Cousijn H, Wallis G, Harrison PJ, Nobre AC (2014) Inter- and intra-individual variability in alpha peak frequency. Neuroimage 92:46–55. 10.1016/j.neuroimage.2014.01.049 24508648 PMC4013551

[B22] Harkins KD, Xu J, Dula AN, Li K, Valentine WM, Gochberg DF, Gore JC, Does MD (2016) The microstructural correlates of T1 in white matter. Magn Reson Med 75:1341–1345. 10.1002/mrm.25709 25920491 PMC4624612

[B23] Hindriks R, Woolrich M, Luckhoo H, Joensson M, Mohseni H, Kringelbach ML, Deco G (2015) Role of white-matter pathways in coordinating alpha oscillations in resting visual cortex. Neuroimage 106:328–339. 10.1016/j.neuroimage.2014.10.057 25449741

[B24] James VS (2002) Independent component analysis: an introduction. Trends Cogn Sci 6:59–64. 15866182 10.1016/s1364-6613(00)01813-1

[B25] Jeffreys H (1961) Theory of probability, Ed 3 Oxford: Oxford University Press.

[B26] Jelescu IO, Veraart J, Fieremans E, Novikov DS (2016) Degeneracy in model parameter estimation for multi-compartmental diffusion in neuronal tissue. NMR Biomed 29:33–47. 10.1002/nbm.3450 26615981 PMC4920129

[B27] Jeurissen B, Tournier JD, Dhollander T, Connelly A, Sijbers J (2014) Multi-tissue constrained spherical deconvolution for improved analysis of multi-shell diffusion MRI data. Neuroimage 103:411–426. 10.1016/j.neuroimage.2014.07.061 25109526

[B28] Klimesch W (1999) EEG alpha and theta oscillations reflect cognitive and memory performance: a review and analysis. Brain Res Brain Res Rev 29:169–195. 10.1016/s0165-0173(98)00056-3 10209231

[B29] Lee IA, Preacher KJ (2013) Calculation for the test of the difference between two dependent correlations with one variable in common [computer software]. Available from http://quantpsy.org.

[B30] Liu Z, de Zwart JA, Yao B, van Gelderen P, Kuo LW, Duyn JH (2012) Finding thalamic BOLD correlates to posterior alpha EEG. Neuroimage 63:1060–1069. 10.1016/j.neuroimage.2012.08.025 22986355 PMC3472152

[B31] Linkenkaer-Hansen K, Nikulin VV, Palva S, Ilmoniemi RJ, Palva JM (2004) Prestimulus oscillations enhance psychophysical performance in humans. J Neurosci 24:10186–10190. 10.1523/JNEUROSCI.2584-04.2004 15537890 PMC6730198

[B32] MacLean MH, Arnell KM, Cote KA (2012) Resting EEG in alpha and beta bands predicts individual differences in attentional blink magnitude. Brain Cogn 78:218–229. 10.1016/j.bandc.2011.12.010 22281183

[B33] Martino J, García-Porrero JA (2013) Wernicke perpendicular fasciculus and vertical portion of the superior longitudinal fasciculus: in reply. Neurosurgery 73:E382–E383. 10.1227/01.neu.0000430303.56079.0e 23624415

[B34] McCunn P, Gilbert KM, Zeman P, Li AX, Strong MJ, Khan AR, Bartha R (2019) Reproducibility of neurite orientation dispersion and density imaging (NODDI) in rats at 9.4 Tesla. PLoS One 14:e0215974. 10.1371/journal.pone.0215974 31034490 PMC6488046

[B35] Mezer A, Yeatman JD, Stikov N, Kay KN, Cho NJ, Dougherty RF, Perry ML, Parvizi J, Hua le H, Butts-Pauly K, Wandell BA (2013) Quantifying the local tissue volume and composition in individual brains with magnetic resonance imaging. Nat Med 19:1667–1672. 10.1038/nm.3390 24185694 PMC3855886

[B36] Mezer A, Rokem A, Berman S, Hastie T, Wandell BA (2016) Evaluating quantitative proton-density-mapping methods. Hum Brain Mapp 37:3623–3635. 10.1002/hbm.23264 27273015 PMC6204063

[B37] Minami S, Amano K (2017) Illusory jitter perceived at the frequency of alpha oscillations. Curr Biol 27:2344–2351.e4. 10.1016/j.cub.2017.06.033 28756954

[B38] Mollink J, Kleinnijenhuis M, Cappellen van Walsum AM, Sotiropoulos SN, Cottaar M, Mirfin C, Heinrich MP, Jenkinson M, Pallebage-Gamarallage M, Ansorge O, Jbabdi S, Miller KL (2017) Evaluating fibre orientation dispersion in white matter: comparison of diffusion MRI, histology and polarized light imaging. Neuroimage 157:561–574. 10.1016/j.neuroimage.2017.06.001 28602815 PMC5607356

[B39] Ogawa S, Takemura H, Horiguchi H, Terao M, Haji T, Pestilli F, Yeatman JD, Tsuneoka H, Wandell BA, Masuda Y (2014) White matter consequences of retinal receptor and ganglion cell damage. Invest Ophthalmol Vis Sci 55:6976–6986. 10.1167/iovs.14-14737 25257055 PMC4215745

[B40] Oishi H, Takemura H, Aoki SC, Fujita I, Amano K (2018) Microstructural properties of the vertical occipital fasciculus explain the variability in human stereoacuity. Proc Natl Acad Sci USA 115:12289–12294. 10.1073/pnas.1804741115 30429321 PMC6275509

[B41] Oostenveld R, Fries P, Maris E, Schoffelen JM (2011) FieldTrip: open source software for advanced analysis of MEG, EEG, and invasive electrophysiological data. Comput Intell Neurosci 2011:156869. 10.1155/2011/156869 21253357 PMC3021840

[B42] Pajevic S, Basser PJ, Fields RD (2014) Role of myelin plasticity in oscillations and synchrony of neuronal activity. Neuroscience 276:135–147. 10.1016/j.neuroscience.2013.11.007 24291730 PMC4037390

[B43] Palva S, Palva JM (2007) New vistas for alpha-frequency band oscillations. Trends Neurosci 30:150–158. 10.1016/j.tins.2007.02.001 17307258

[B44] Panesar SS, Belo JTA, Yeh F-C, Fernandez-Miranda JC (2019) Structure, asymmetry, and connectivity of the human temporo-parietal aslant and vertical occipital fasciculi. Brain Struct Funct 224:907–923. 10.1007/s00429-018-1812-0 30542766 PMC7026858

[B45] Renauld E, Descoteaux M, Bernier M, Garyfallidis E, Whittingstall K (2016) Semi-automatic segmentation of optic radiations and LGN, and their relationship to EEG alpha waves. PLoS One 11:e0156436 10.1371/journal.pone.0156436 27383146 PMC4934857

[B46] Samaha J, Postle BR (2015) The speed of alpha-band oscillations predicts the temporal resolution of visual perception. Curr Biol 25:2985–2990. 10.1016/j.cub.2015.10.007 26526370 PMC4654641

[B47] Sampaio-Baptista C, Johansen-Berg H (2017) White matter plasticity in the adult brain. Neuron 96:1239–1251. 29268094 10.1016/j.neuron.2017.11.026 PMC5766826

[B48] Schilling KG, Janve V, Gao Y, Stepniewska I, Landman BA, Anderson AW (2018) Histological validation of diffusion MRI fiber orientation distributions and dispersion. Neuroimage 165:200–221. 10.1016/j.neuroimage.2017.10.046 29074279 PMC5732036

[B49] Setsompop K, Cohen-Adad J, Gagoski BA, Raij T, Yendiki A, Keil B, Wedeen VJ, Wald LL (2012) Improving diffusion MRI using simultaneous multi-slice echo planar imaging. Neuroimage 63:569–580. 10.1016/j.neuroimage.2012.06.033 22732564 PMC3429710

[B50] Sherbondy AJ, Dougherty RF, Napel S, Wandell BA (2008a) Identifying the human optic radiation using diffusion imaging and fiber tractography. J Vis 8:12.1–11. 10.1167/8.10.12 19146354 PMC2759943

[B51] Sherbondy AJ, Dougherty RF, Ben-Shachar M, Napel S, Wandell BA (2008b) ConTrack: finding the most likely pathways between brain regions using diffusion tractography. J Vis 8:1–16. 10.1167/8.9.15 PMC2696074 18831651

[B52] Sokoliuk R, VanRullen R (2013) The flickering wheel illusion: when α rhythms make a static wheel flicker. J Neurosci 33:13498–13504. 10.1523/JNEUROSCI.5647-12.2013 23946408 PMC6705156

[B53] Stikov N, Campbell JS, Stroh T, Lavelée M, Frey S, Novek J, Nuara S, Ho MK, Bedell BJ, Dougherty RF, Leppert IR, Boudreau M, Narayanan S, Duval T, Cohen-Adad J, Picard PA, Gasecka A, Côté D, Pike GB (2015) In vivo histology of the myelin g-ratio with magnetic resonance imaging. Neuroimage 118:397–405. 10.1016/j.neuroimage.2015.05.023 26004502

[B54] Takemura H, Pestilli F, Weiner KS, Keliris GA, Landi SM, Sliwa J, Ye FQ, Barnett MA, Leopold DA, Freiwald WA, Logothetis NK, Wandell BA (2017) Occipital white matter tracts in human and macaque. Cereb Cortex 27:3346–3359. 10.1093/cercor/bhx070 28369290 PMC5890896

[B55] Takemura H, Ogawa S, Mezer AA, Horiguchi H, Miyazaki A, Matsumoto K, Shikishima K, Nakano T, Masuda Y (2019) Diffusivity and quantitative T1 profile of human visual white matter tracts after retinal ganglion cell damage. Neuroimage Clin 23:101826. 10.1016/j.nicl.2019.101826 31026624 PMC6482365

[B56] Thut G, Nietzel A, Brandt SA, Pascual-Leone A (2006) Alpha-band electroencephalographic activity over occipital cortex indexes visuospatial attention bias and predicts visual target detection. J Neurosci 26:9494–9502. 10.1523/JNEUROSCI.0875-06.2006 16971533 PMC6674607

[B57] Tournier JD, Calamante F, Connelly A (2012) MRtrix: diffusion tractography in crossing fiber regions. Int J Imaging Syst Technol 22:53–66. 10.1002/ima.22005

[B58] Tzourio-Mazoyer N, Landeau B, Papathanassiou D, Crivello F, Etard O, Delcroix N, Mazoyer B, Joliot M (2002) Automated anatomical labeling of activations in SPM using a macroscopic anatomical parcellation of the MNI MRI single-subject brain. Neuroimage 15:273–289. 10.1006/nimg.2001.0978 11771995

[B59] van Dijk H, Schoffelen JM, Oostenveld R, Jensen O (2008) Prestimulus oscillatory activity in the alpha band predicts visual discrimination ability. J Neurosci 28:1816–1823. 10.1523/JNEUROSCI.1853-07.2008 18287498 PMC6671447

[B60] Veen BDV, Drongelen WV, Yuchtman M, Suzuki A (1997) Localization of brain electrical activity via linearly constrained minimum variance spatial filtering. IEEE Trans Biomed Eng 44:867–880. 10.1109/10.623056 9282479

[B61] Wake H, Ortiz FC, Woo DH, Lee PR, Angulo MC, Fields RD (2015) Nonsynaptic junctions on myelinating glia promote preferential myelination of electrically active axons. Nat Commun 6:7844. 26238238 10.1038/ncomms8844 PMC4532789

[B62] Wang L, Mruczek REB, Arcaro MJ, Kastner S (2015) Probabilistic maps of visual topography in human cortex. Cereb Cortex 25:3911–3931. 10.1093/cercor/bhu277 25452571 PMC4585523

[B63] Wang N, Zhang J, Cofer G, Qi Y, Anderson RJ, White LE, Johnson GA (2019) Neurite orientation dispersion and density imaging of mouse brain microstructure. Brain Struct Funct 224:1797–1813. 10.1007/s00429-019-01877-x 31006072 PMC6565502

[B64] Weiner KS, Yeatman JD, Wandell BA (2017) The posterior arcuate fasciculus and the vertical occipital fasciculus. Cortex 97:274–276. 10.1016/j.cortex.2016.03.012 27132243 PMC6760835

[B65] Wetzels R, Wagenmakers E-J (2012) A default Bayesian hypothesis test for correlations and partial correlations. Psychon Bull Rev 19:1057–1064. 10.3758/s13423-012-0295-x 22798023 PMC3505519

[B66] Yeatman JD, Dougherty RF, Myall NJ, Wandell BA, Feldman HM (2012) Tract profiles of white matter properties: automating fiber-tract quantification. PLoS One 7:e49790. 10.1371/journal.pone.0049790 23166771 PMC3498174

[B67] Yeatman JD, Weiner KS, Pestilli F, Rokem A, Mezer A, Wandell BA (2014) The vertical occipital fasciculus: a century of controversy resolved by in vivo measurements. Proc Natl Acad Sci USA 111:E5214–E5223. 10.1073/pnas.1418503111 25404310 PMC4260539

[B68] Zhang H, Schneider T, Wheeler-Kingshott CA, Alexander DC (2012) NODDI: practical in vivo neurite orientation dispersion and density imaging of the human brain. Neuroimage 61:1000–1016. 10.1016/j.neuroimage.2012.03.072 22484410

